# Impact of smart phone use on adolescence health in India

**DOI:** 10.6026/973206300191090

**Published:** 2023-11-30

**Authors:** Mahalakshmi B, Sivasubramanian N, Dhobi Nirali Dasharathbhai, Gnanadesigan Ekambaram

**Affiliations:** 1 Department of Paediatric Nursing, Nootan College of Nursing, Sankalchand Patel University, Visnagar, Gujarat - 384315, India; 2 Department of Psychiatric Nursing, Nootan College of Nursing, Sankalchand Patel University, Visnagar, Gujarat - 384315, India; 3 Department of Community health Nursing, Nootan College of Nursing, Sankalchand Patel University, Visnagar, Gujarat - 384315, India; 4 Department of Physiology, Nootan Medical College and Research Centre, Sankalchand Patel University, Visnagar, Gujarat - 384315; India

**Keywords:** Codex knowledge, impact, smart phone, health dimensions

## Abstract

Smart phone use is on the rise globally, which may have an impact on people's health. The second-largest country in terms of mobile phone usage is India. However, there aren't many studies that have been done in India to evaluate its health impacts. Therefore, it is of interest to assess the effectiveness of codex on the impact of smart phone use on various dimensions of health status among adolescence. The pre-test mean score for knowledge regarding the impact of smart phones on physical health was 1.92, while the post-test mean score was 3.75. The pre-test and post-test standard deviations were 0.91 and 0.93, respectively, and the mean deviation score was the 't'-value that was 11.000 and Significant at the p<0.001*** level. The research was conducted in higher secondary school, and 60 students participated. The research design was a pre-experimental design; a self-administered questionnaire was used to assess the knowledge of Students on the Impact of mobile phone use on various dimensions of health. Most people, on average, spend 3 hours and 15 minutes on their phones each day. As adolescents are engaged in smartphone use, it disturbs their sleep patterns, adversely impacting their short-term memory

## Background:

Every aspect of existence requires communication. It doesn't matter if one is at work, at school, on the job, or just out socializing. Nevertheless, it could involve anything from making phone calls or sending emails to making presentations or drafting reports. A mobile phone is an easy-to-use, portable device. Without complex technology or specialized skills, it is simple to use. Students' use of smartphones is on the rise, and this has led to issues including lack of focus, indiscipline, poor academic performance, and limited class engagement, which kept the average difference for students who experienced affected at 22.5 percentages. [1] A smartphone has the functions of a mobile phone and the Internet. Smartphones provide qualitatively different services. Smartphones are used by young people to learn online courses, gain knowledge, express themselves, connect with friends and relatives, use various apps, and explore information, while senior users use them to make video calls to their distant children and for better communication. Although smartphones provide many benefits, we also need to be aware of their drawbacks, one of the most concerning of being smartphone addiction. A study suggests that among adolescents, smartphone use may result in learning opportunities being unequal. Finally, it is demonstrated that smartphone behavior is the mediating variable impacting academic achievement after a discussion of whether or not it is a mediator of performance [2]. Less access to smartphones may have a negative impact on academic achievement and learning effectiveness. These kinds of people encounter social, psychological, and health issues [3]. There is a need for public awareness campaigns concerning smartphone addiction and how it affects both physical and mental health [4]. There are 600 million smartphone users in India [5]. Regarding body image dissatisfaction, depression, social anxiety, emotional abuse, and neglect were positively correlated with problematic smartphone use both directly and indirectly [6]. Smartphone usage has many other problems too, for example, emotional abuse and neglect were directly and indirectly related positively to problematic smartphone use via body image dissatisfaction, depression, and social anxiety [7]. In addition to providing enjoyment and lowering pain and stress, using a smartphone can also result in failure to limit usage despite detrimental effects on one's especially adolescent’s health, finances, physical well-being, and relationships with others [8]. 83.9% of people were found to use smartphones. It was linked to factors like age, place of residence, behavior, use of a hands-free device, and parents' educational and financial status. 37% of people are said to be addicted to their smartphones. It was discovered to be related to age, site of residence, educational institution, length of smartphone use, daily hours of use, the belief that mobile use is unhealthy, and parents' educational and financial status [9]. It is also been proved that significant correlations between excessive smartphone use and depression, anxiety disorders, excessive internet and game use, alcoholism, and nicotine dependency disorders were also found [10]. Thus it has many advantages and disadvantages; the researcher was trying to reduce smartphone addiction among adolescents. Therefore, it is of interest to evaluate the efficiency of Codex's understanding of the effects of mobile phone users.

## Methodology:

The methodology of research organizes all the components of the study in a way that is most likely to lead to valid answers to the sub-problems that have been posed. This study aims at finding out the impact of mobile phone use on various dimensions of students 'health status. In this study proposed stratified random sampling design was used, population was 60 students aged 16-19 years, from Visnagar, Mehsana, Gujarat. The research design selected for the study was one group pre-post test design (01 X 02), which belongs, to the pre-experimental design [11, 12]. Pre-test was done followed by the administration of a self-administered questionnaire and then conducted post-test for the same group after 7 days. Study was approved by institutional ethics committee, and all subjects gave their informed consent after the study methods and goal were explained in local language (Gujarati). The experiments followed the amended Helsinki Declaration of 1975 that was revised in 2013.A self-administered questionnaire on Codex on Knowledge regarding mobile phone impact was used. Totally it has 30 questions with 4 domains of physical, social, psychological, and spiritual. The scoring of the pattern is as follows

[1] Each correct answer scores one mark

[2] Each wrong answer scores a zero mark

[3] Total maximum marks is 30

[4] Minimum mark is 0

The score pattern was: 1-10 inadequate knowledge, 10-20 Moderate knowledge, 20-30 adequate knowledge.

## Statistical analysis:

SPSS (Statistical Package for Social Sciences) was used to conduct the statistical analysis (version 17.0) of Descriptive and inferential statistics. The Student's t-test is used to examine the results, which are given as mean standard deviation (SD). A statistically significant P-value of 0.05 was used.

## Results:

**Figure 1:**
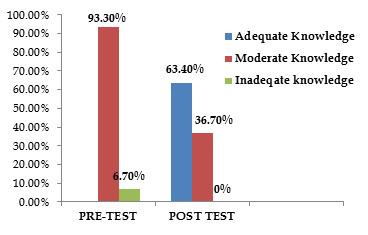
The level of knowledge in pre-and post-test among adolescents

The present study showed that 60 participants majority were male students, and all are unmarried. It was found that most participants were belongs to nuclear family and among them 35 % had monthly family income between Rs 30000-50000. It was also found that some participants had other electronic devices like laptop. Majority of participants belongs to semi-urban region. Table 1 and Figure 1 show distribution of samples according to the level of knowledge regarding the impact of smart phone use and shows reports of pre & post-test in frequency & percentage level. 93.3 percent people show moderate knowledge regarding impact of mobile smart phone usage. Maximum participation was from the age group of 19 years, which accounts for 45% of total participation. Out of total participants 52.2% were male students and 47% were female students. Most of the people are college students and are found to be using 4G internet connectivity over the mobile network, Wi-Fi & broadband connection. Participants having inadequate knowledge regarding impact of smartphone use belong to rural areas. Table 2 shows mean values of pre-test & post-test of impact of smart phone uses before and after administration of codex. It shows statistically significant (p value <0.001).

## Discussion:

The main conclusion drawn from the present study was that most of the main focus of the study was to codex on knowledge regarding the impact of mobile phone use on various dimensions of health status among adolescence; data were collected from 60 samples through proportionate stratified random sampling technique. According to recent research, psychological interventions can be successful in lowering problematic Internet and smartphone use. The results must be viewed as tentative, though, because there aren't many studies in those sectors [13]. Children's lack of social networks may prevent them from feeling supported and engaging in comfortable social interactions offline, which may increase their need to flee to their smartphones [14, 15]. The implications are given on the various aspects like nursing education, nursing practice, and nursing administrations and it also gives insight into the future studies. A study's findings confirm the value of a Health Belief Model-based educational intervention for preventing and reducing smartphone addiction [16]. One more study also supported that the peer education model was effective in reducing smartphone addiction in adolescents [17]. Many studies show that there is a lack of knowledge regarding the impact of mobile phone use on health status among students. Codex is the best method to improve knowledge of the impact of mobile phone use on health status among students. Despite its effects on human physiology, psychology, and social behavior, smartphone addiction has not yet been recognized as a problem on a global scale. Spain has the most outside of Asia; China and South Korea have produced the majority of studies in this field. Additionally, due to the ease of sampling, the majority of the research participants were probably students. Future research may concentrate on smartphone addiction among people of different ages as cell phones become more common among older individuals [18, 19]. Users of mobile phones have an emotional bond with them that causes them to feel as though they cannot exist without a cell phone [20]. In the current study, the knowledge assessed was inadequate and negative when assessed. So, the codex was given to maintain adequate knowledge.

## Conclusion:

Most people, on average, spend 3 hours and 15 minutes on their phones each day. As adolescents are engaged in smartphone use, it disturbs their sleep patterns, adversely impacting their short-term memory, resulting in negativity, distress, less emotional recovery, and spoiling spirituality. Especially the school going adolescents are severely affected by their academic activity. Thus, the researcher tried to reduce smartphone addiction by giving Codex on mobile phone use to reduce smartphone addiction and found it was more effective.

## Figures and Tables

**Table 1:** Distribution of samples according to the level of knowledge regarding the impact of smart phone use

**Level of Knowledge**	**Grading**	**Pre-test**		**Post-test**	
		**Frequency**	**Percentage**	**Frequency**	**Percentage**
Adequate Knowledge	>20	-	-	38	63.4
Moderate Knowledge	10-20	56	93.3	22	36.7
Inadequate Knowledge	<10	4	6.7	-	-
N= 60

**Table 2:** The impact of smart phone use before and after administration of codex

**Time**	**Mean**	**SD**	**Mean difference**	**t value**	**p-value**
Pre-test	11.9	2.2	9.23	26.47	<0.001***
Post-test	21.13	2.17			
N=60

## References

[1] Feitosa AGC (2019). International Journal for Innovation Education and Research.

[2] Wang JC (2023). Education and Information Technologies.

[3] Abi-Jaoude E (2020). Canadian medical association journal..

[4] Ratan ZA (2021). Int J Environ Res Public Health..

[5] Heron D, Shapira NA (2003). Current Psychiatry.

[6] Ayhualem S (2021). PLOS ONE.

[7] Shaffer HJ. (1996). Journal of Gambling Studies.

[8] Ting CH, Chen YY (2020). Adolescent addiction.

[9] Bhanderi DJ (2021). Indian J Community Med.

[10] Soobin Jo (2021). Psychiatry Research,.

[11] Mahalakshmi B (2022). Bioinformation.

[12] Mahalakshmi B (2023). Bioinformation.

[13] Augner C (2021). Australian and New Zealand journal of Psychiatry.

[14] Ihm J (2018). Journal of Behavioural Addiction..

[15] Gangadharan N (2022). Cureus..

[16] Koshgofter M (2019). International journal of Paediatrics.

[17] Avci D (2023). Health education research.

[18] Cha SS, Seo BK (2018). Health Psychol Open..

[19] Sivasubramanian N (2022). Bioinformation.

[20] Babadi-Akashe Z (2014). Addict health.

